# Supplementary material for: The limits of human mobility traces to predict the spread of COVID-19: A transfer entropy approach
Source: PNAS Nexus. 2023 Sep 14;2(10):pgad302. doi: 10.1093/pnasnexus/pgad302 (PMC10558401; doi:10.1093/pnasnexus/pgad302)
Supplement: pgad302_Supplementary_Data [file pgad302_supplementary_data.pdf]

Supplementary Information for
The limits of human mobility traces to predict the spread of COVID-19: a transfer entropy approach

This is the supplementary information for the manuscript *The limits of human mobility traces to predict the spread of COVID-19: a transfer entropy approach*, containing supplementary tables and figures.

1 Definition of mobility metrics

We first describe the raw data sources provided by Meta and then the data processing we applied to compute the time series for the transfer entropy analysis.

1.1 Raw data sources

We collected the following datasets that were publicly released by Meta since the beginning of the COVID-19 pandemic, in Austria, France, Italy, and Spain:

- **Movement range maps**. It reports the number of users who moved between any two 16-level Bing tiles, with an 8-hour frequency.
- **Users' population.** It reports the number of active users in each tile with an 8-hour frequency. The tile resolution is 4800 x 4800 m^2.
- **Colocation maps.** It estimates the probability that, given any two administrative regions, p_1 and p_2, a randomly chosen user from p_1 and a randomly chosen user from p_2 are simultaneously located in the same place during a randomly chosen minute in a given week **?**. The dataset also reports the *number of users* in p_1 and p_2.
- **Stay put**. It reports for a given administrative region the daily percentage of users staying put within a single location, defined at the 16-level Bing tile.

We formalize the description of the above datasets with the notation described in Table S1:

Dataset name	$X_{s,t}$	spatial resolution	temporal resolution
**population users**	$N^{(pop)}_{t,h}$	t: tile (4800 x 4800 m^2)	h: 8 hour
**movement between tiles**	$M_{(t1,t2),h}$	(t1,t2): tile pair (600 x 600 m^2)	h: 8 hour
**colocation probability**	$P_{p,w}$	p: province	w : week
**colocation users**	$N^{(coloc)}_{p,w}$	p: province	w: week
**stay put**	$S_{r,d}$	r: region	d: day

Table S1: Summary of raw data sources as time series records $X_{s,t}$, where s denotes the spatial resolution and t the temporal resolution.

1.2 Aggregation of raw data

original data	spatial aggregation	temporal aggregation	aggregated data	name
$N^{(pop)}_{t,h}$	$\sum(t \in p)$	h interpolation and mean $(h \in w)$	$N^{(pop)}_{p,w}$	**province population users**
$M_{(t1,t2),h}$	$\sum\ (t_1, t_2) \in p, t_1 = t_2$	mean $(h \in w)$	$M^{(within)}_{p,w}$	**within tile province movement**
$M_{(t1,t2),h}$	$\sum\ (t_1, t_2) \in p, t_1 \neq t_2$	mean $(h \in w)$	$M^{(between)}_{p,w}$	**between tiles province movement**
$S_{r,d}$	$\forall p \in r \quad r = p$	mean $(d \in w)$	$S_{p,w}$	**province stay put**

Table S2: Aggregation of data sources described in Table S1, to generate our metrics of interest.

We then processed the raw data sources of Table S1 to obtain a set of time series having the same spatiotemporal resolution, that is weekly, at the NUTS3 scale. Results of the aggregation process are described in Table S2. More in detail:

- **Province users population.** (1) we performed a spatial aggregation by summing the population of tiles belonging to province p, thus obtaining a population at a (province, hour) level: $N^{(pop)}_{p,h}$. (2) we performed a linear interpolation of the temporal gaps that were present in $N^{(pop)}_{p,h}$ (3) we performed a temporal aggregation by averaging in each province, the 8h population records within a week.
- **Within tile province movement** (1) we first performed a temporal aggregation by averaging $M_{(t1,t2),h}$ for each pair $(t1, t2)$ over a week and obtaining $M_{(t1,t2),w}$ (2) we then performed a spatial feature joining and assigned each pair $(t1, t2)$ to the corresponding provinces $(p1, p2)$ (3) from $M_{(t1,t2),w}$ we obtained a **within tile province movement** $M^{(within)}_{p,w}$, that is the sum of movements which occurred in the same province p and within the same tile.

- **Between tiles province movement** in the pipeline above, from step (3) we obtain a **between tile province movement** $M_{p,w}^{(between)}$, that is the sum of movements which occurred in the same province p and between two different tiles, $(t1, t2)$. By definition, the sum $M_{p,w}^{(between)} + M_{p,w}^{(within)}$ represents the total volume of movements in a province, in a week.
- **Province stay put** (1) we performed a temporal aggregation on a weekly scale by performing the average and obtaining $S_{r,w}$ (2) we assign to each province p the regional stay-put time series $S_{r,w}$ such that $p \in r$.

1.3 Computation of movement and contact rate

We finally computed our metrics of interest, movement, and contact rates, as follows. The short-range movement rate is defined as:

$$M_{p,w}^{s} = \frac{M_{p,w}^{(within)}}{N_{p,w}^{(pop)}} \tag{1}$$

that is the proportion of users who moved within the same tile in a given province, in a given week. The mid-range movement rate is defined as:

$$M_{p,w} = \frac{M_{p,w}^{(between)}}{N_{p,w}^{(pop)}} \tag{2}$$

representing the proportion of users who moved between different tiles in a given province, in a given week. The contact rate is defined as:

$$CR(t)_{p,w} = \hat{P}_{p,w} \cdot N_{p,w}^{(pop)} \tag{3}$$

where $\hat{P}$ denotes the colocation probability corrected by a factor that takes into account the overestimation of colocation probabilities due to the heterogeneous distribution of users across provinces and the presence of a significant fraction of static users in some periods of mobility restrictions.

1.4 Correction to the colocation probability

Colocation maps provided by Meta are defined as the number of colocation events over the number of possible events. This, by design, includes interactions between users staying within the same tile but not having actual contact with other users. For this reason, we estimate the contact rate in each province by removing the contribution due to the users staying put. We explain our approach to estimating such contribution in the following.

Let us start by writing the original colocation probability P as:

$$P = \frac{E}{N^2} \tag{4}$$

where:

- E is the number of colocation events within the province
- N is the number of province colocation users.

The exact formula should be $P = \frac{E}{N(N-1)}$ but as N is large we approximate it to 4. Let us denote $R^{(c)}$ the number of measured colocation events that are due to users who stay put only, then the corrected colocation probability should be written in the following way:

$$\hat{P}_{p,w} = \frac{E - R^{(c)}}{N^2} \tag{5}$$

We estimate $R^{(c)}$ by using the stay-put probability S, which is the probability of a user staying put. Let us call the tile population ratio probability distribution $\{f_{t_l}; t = 1, .., T_l\}$ where T is the number of tiles in a province. This gives us an estimate of the contribution of the users who stay put to the colocation probability, as:

$$R^{(c)} = \sum_{t=1}^{T} N^2 \cdot f_t^2 \cdot S^2. \tag{6}$$

So we rewrite:

$$\hat{P}_{p,w} = P - S^2 \sum_{t=1}^{T_l} f_{t_l}^2 \tag{7}$$

We do not have access to the population of the tiles used for the colocation so we make an approximation using the population distribution given for each tile with dimensions 4800 m × 4800 m. As there are by definition 64 colocation tiles within a single population tile, the expression Eq.7 can be formulated as:

$$\hat{P}_{p,w} = P_{p,w} - S_{p,w}^2 \cdot \sum_{t=1}^{T} 64 \cdot \left(\frac{f_{t,w}^{(p)}}{64} \right)^2 \tag{8}$$

where:

- $f_{t,w}^{(p)} = \frac{N_{t,w}}{N_{p,w}}; t \in p$: tile t population frequency in province p.
- $N_{t,w}$: population at (tile,week) resolution. It is obtained through mean temporal aggregation of $N_{t,h}$ over the week interval denoted by w.
- $N_{p,w}$: population at (province, week) resolution. It is obtained through sum spatial aggregation of $N_{t,w}$ over the tiles belonging to province p.
- T is the number of tiles 4800 m × 4800 m

We can introduce the quantity $Q_{p,w}$ as the sum of squared frequencies of the province tile distribution $Q_{p,w} = \sum_{t \in p} (f_{t,w}^{(p)})^2$, so that, finally:

$$\hat{P}_{p,w} = P_{p,w} - \frac{S_{p,w}^2 \cdot Q_{p,w}}{64} \tag{9}$$

	M^s (%)	M (%)
Austria	99.6 [97.9 – 100]	0.5 [0.0 - 2.1]
France	91.3 [88.2 – 93.7]	8.8 [6.3 – 11.9]
Italy	89.9 [86.4 – 92.8]	10.2 [7.2 – 13.6]
Spain	91.5 [86.1 – 95.1]	8.5 [4.9 – 13.9]

Table S3: **Relative proportion of mobility components in each country.** Each row displays the proportion of movements, as a percentage of the total movements within each province, that are represented by the short-range mobility ($M^s(t)$) and the mid-range mobility ($M(t)$). Each table entry reports the median value and the IQR, computed over all provinces, and all weeks of the study period. Short-range mobility represents the large majority of movements within a province, in all countries.

	coverage consistency	commuting flow
Austria	0.64 [0.45–0.79]	1.05 [0.43–1.69]
France	0.32 [0.23–0.43]	0.30 [0.22–0.51]
Italy	0.63 [0.42–0.77]	0.21 [0.12–0.29]
Spain	0.86 [0.68–0.91]	0.08 [0.05–0.10]

Table S4: **Coverage consistency and commuting flow distributions by country.** Each table entry reports the median value and the IQR computed over all provinces, in each country, considered in the study.

	$\rightarrow C(t)(\%)$		$\rightarrow D(t)(\%)$		$\rightarrow R_t(t)(\%)$	
l (weeks)	$M^s(t)$	$M(t)$	$M^s(t)$	$M(t)$	$M^s(t)$	$M(t)$
2	NA	5 (2)	4	NA	3 (1)	2
3	4 (0)	5 (2)	4 (1)	NA	NA	3
4	4 (1)	4 (0)	4 (1)	NA	3 (0)	3
5	4 (1)	4 (1)	4 (2)	NA	5 (1)	3
6	4 (2)	4 (1)	4 (1)	NA	5 (2)	NA
7	9 (4)	NA	5 (2)	4 (1)	5 (2)	NA
8	8 (3)	NA	5 (4)	5 (1)	4 (1)	5

Table S5: **NETE results across regions in Italy.** The table shows the average relative explanation added by source time series, with respect to past knowledge of the target only. Only regions having a statistically significant NETE are considered. Numbers in parenthesis report the standard deviation computed over all regions for which the NETE was statistically significant.

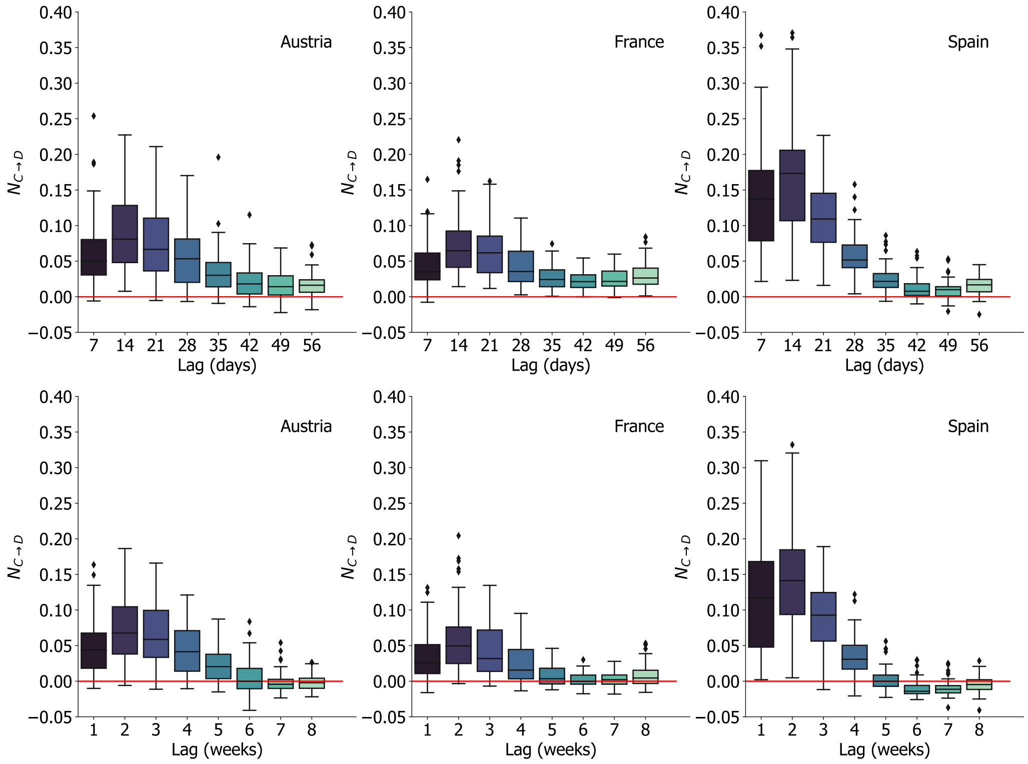

Figure S1: **Comparison of NETE values computed on weekly and daily time series.** $N_{C \to D}$ computed between time series data collected on a weekly time scale (bottom row) and a daily one (top row). Daily time series were available only for Austria, France and Spain.

	$\to C(t)(\%)$		$\to D(t)(\%)$		$\to R_t(t)(\%)$	
l (weeks)	$M^s(t)$	$M(t)$	$M^s(t)$	$M(t)$	$M^s(t)$	$M(t)$
2	0	17	6	0	11	6
3	17	17	11	0	0	6
4	22	11	17	0	11	6
5	28	11	17	0	33	6
6	33	11	28	0	61	0
7	11	0	28	17	50	0
8	11	0	17	17	28	6

Table S6: **Percentage of statistically significant NETE values across regions in Italy**. This table shows the percentage of regions, in Italy, in which the NETE is statistically significant ($p < 0.01$) for lags (l) from 2 to 8 weeks.

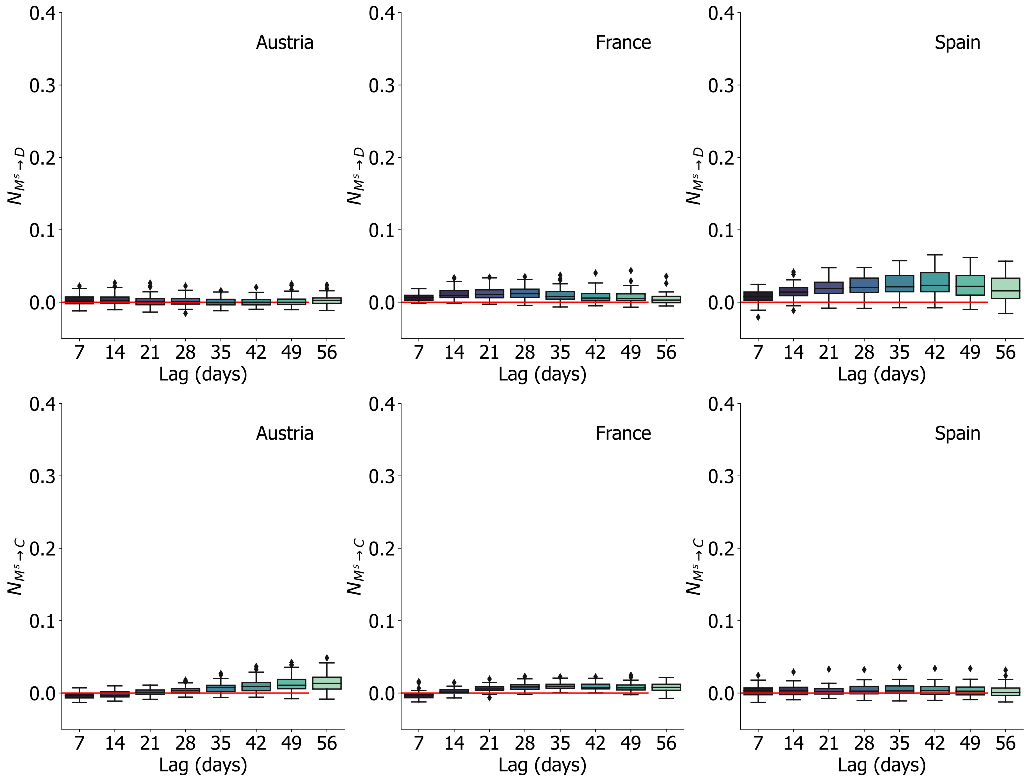

Figure S2: **NETE values computed on daily time series.** $N_{M^s \to D}$ computed between short-range movements and deaths (top row) and $N_{M^s \to C}$ computed between short-range movement and cases (bottom row). Daily time series were available only for Austria, France and Spain.

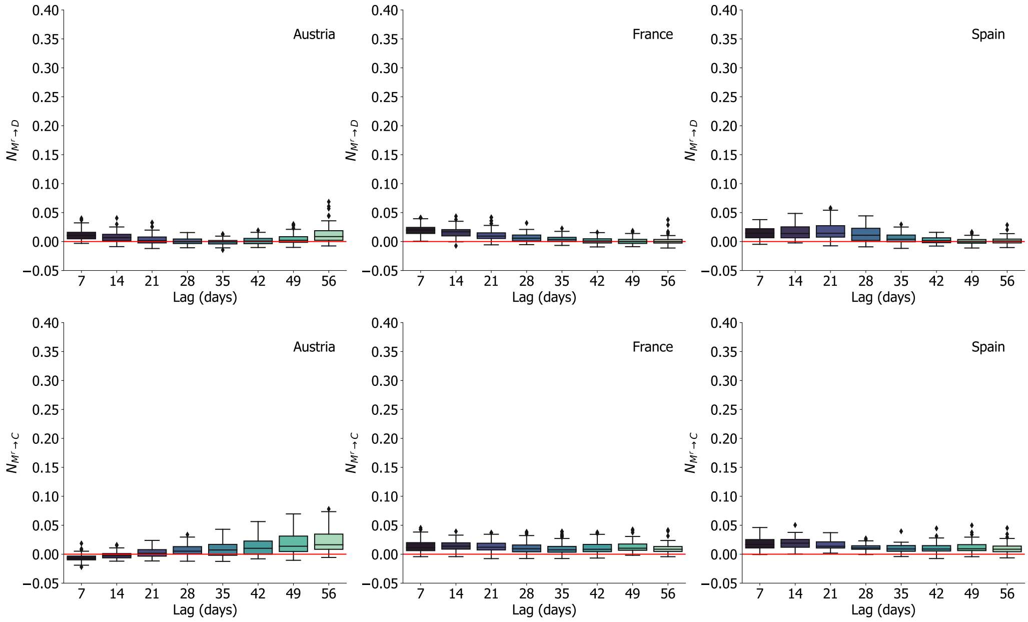

Figure S3: **NETE values computed on daily time series.** $N_{M^r \to D}$ computed between change in residential stay and deaths (top row) and $N_{M^r \to C}$ computed between change in residential stay and cases (bottom row). Daily time series were available only for Austria, France and Spain.

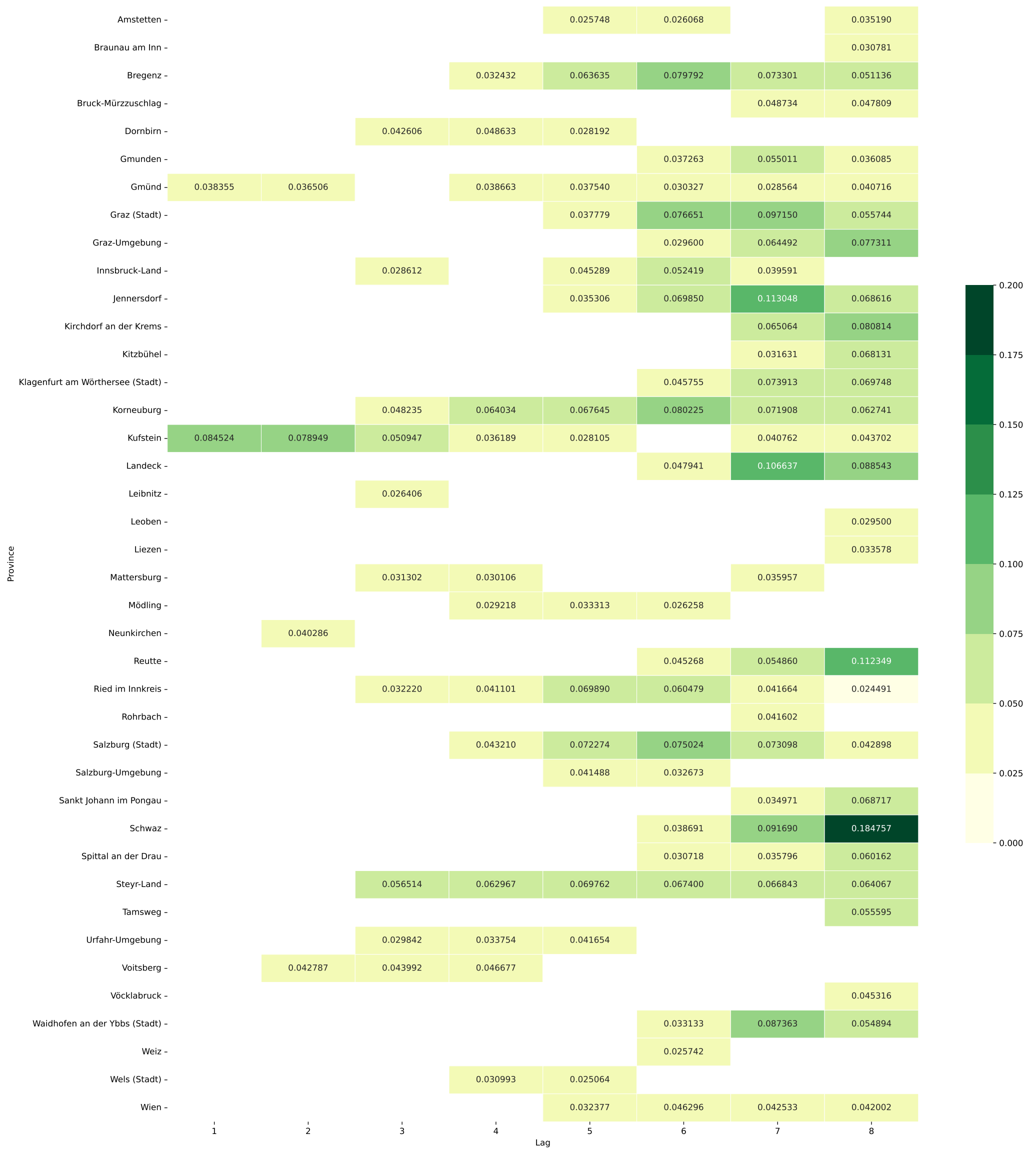

Figure S4: **NETE values from contact rates to deaths in Austria.** Only statistically significant values are shown (p-value< 0.01).

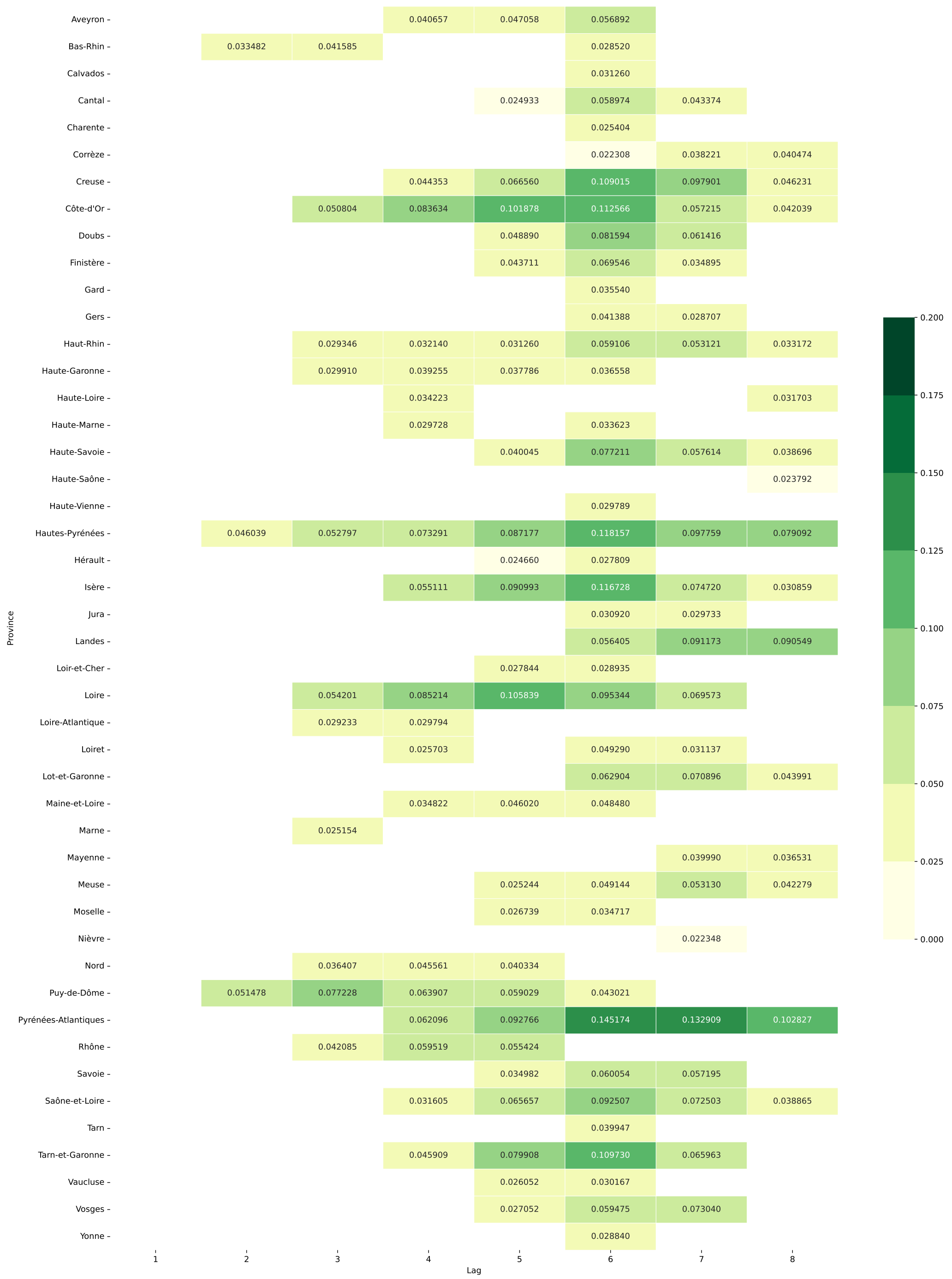

Figure S5: **NETE values from contact rates to deaths in France.** Only statistically significant values are shown (p-value< 0.01).

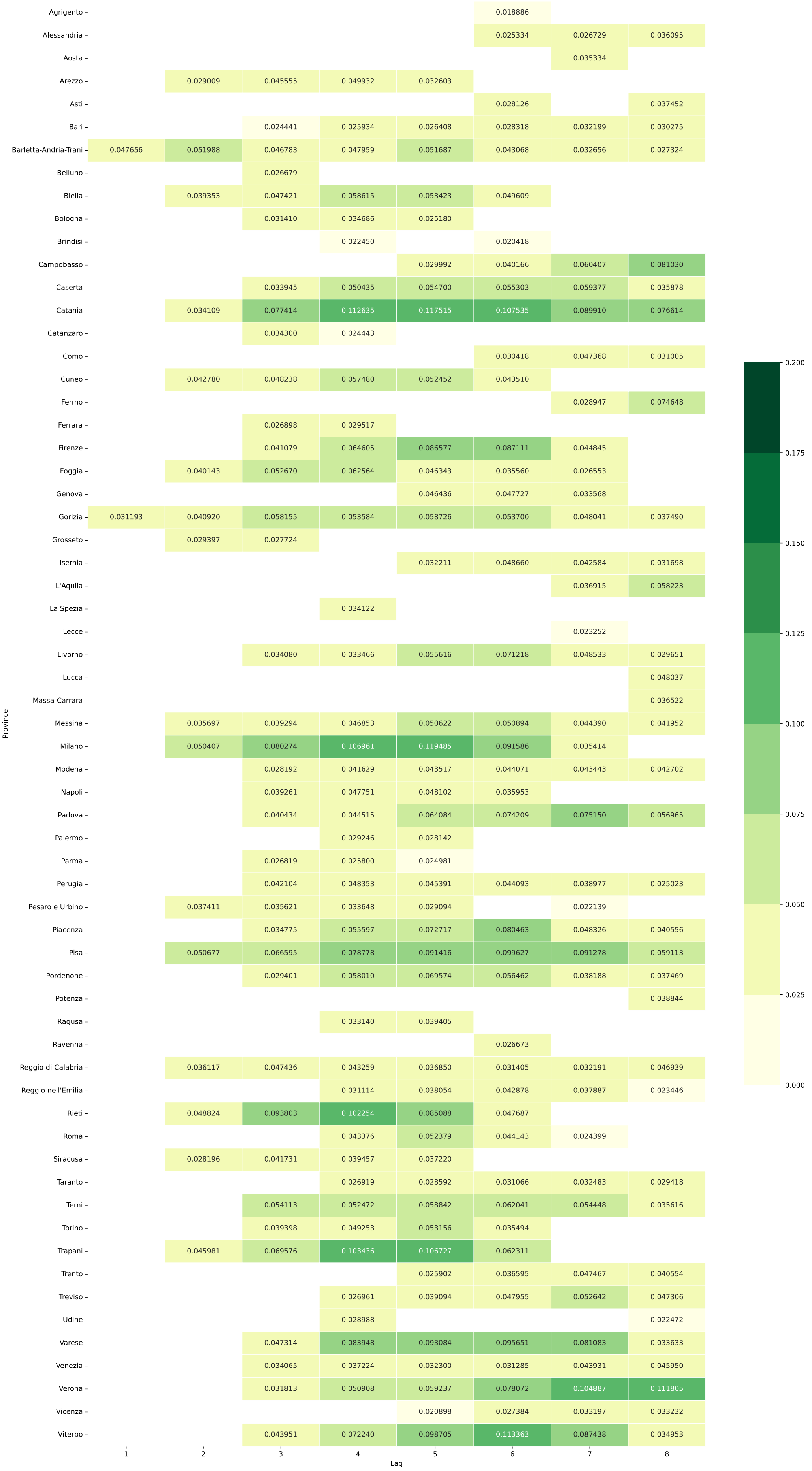

Figure S6: **NETE values from contact rates to deaths in Italy.** Only statistically significant values are shown (p-value< 0.01).

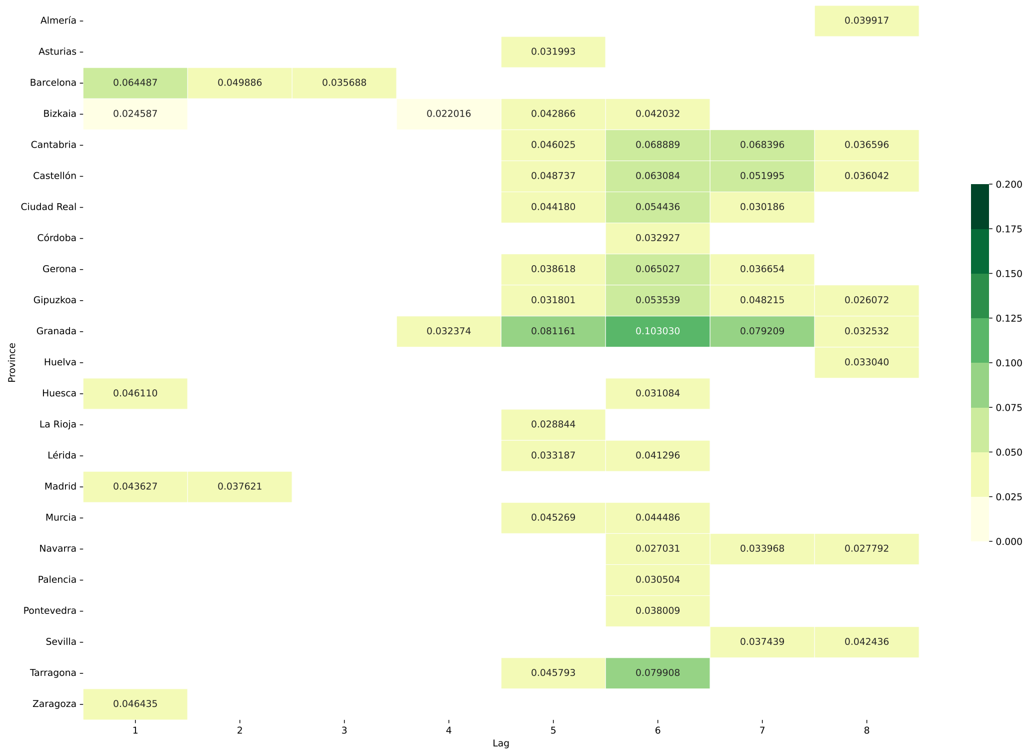

Figure S7: **NETE values from contact rates to deaths in Spain.** Only statistically significant values are shown (p-value< 0.01).

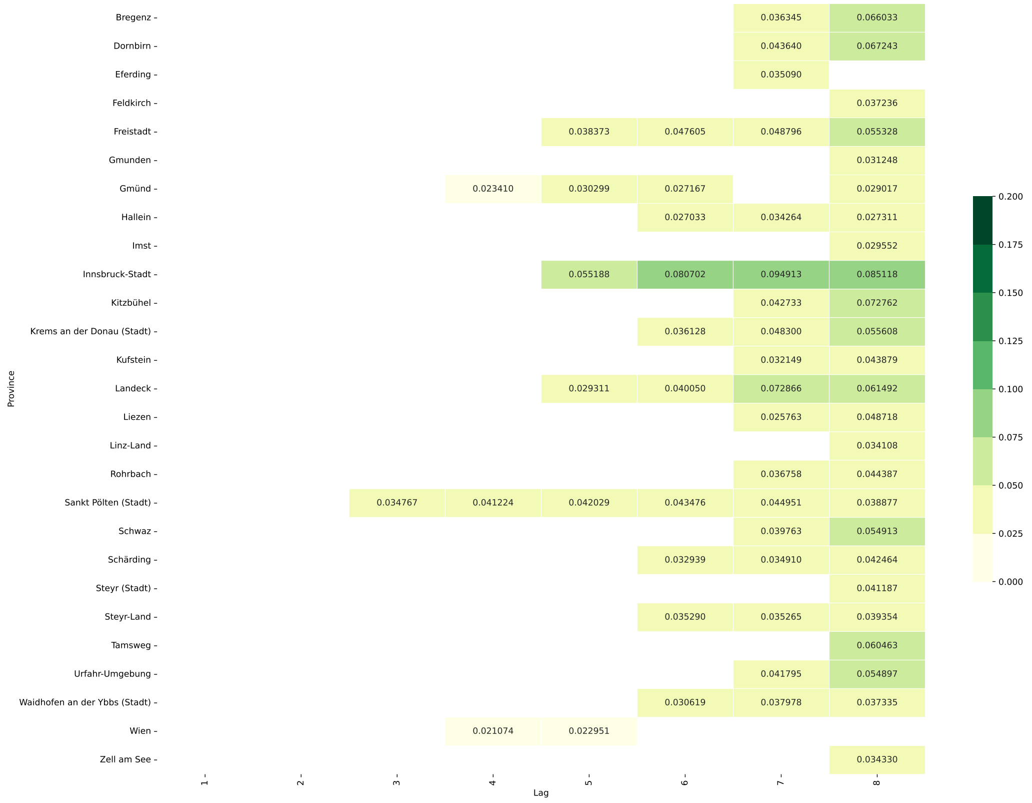

Figure S8: **NETE values from movements to deaths in Austria.** Only statistically significant values are shown (p-value< 0.01).

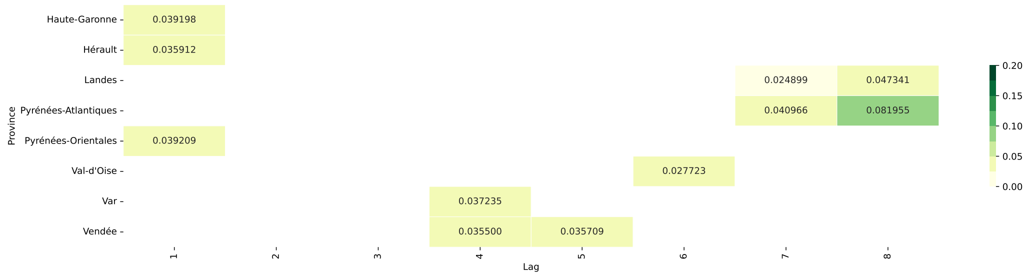

Figure S9: **NETE values from movements to deaths in France.** Only statistically significant values are shown (p-value< 0.01).

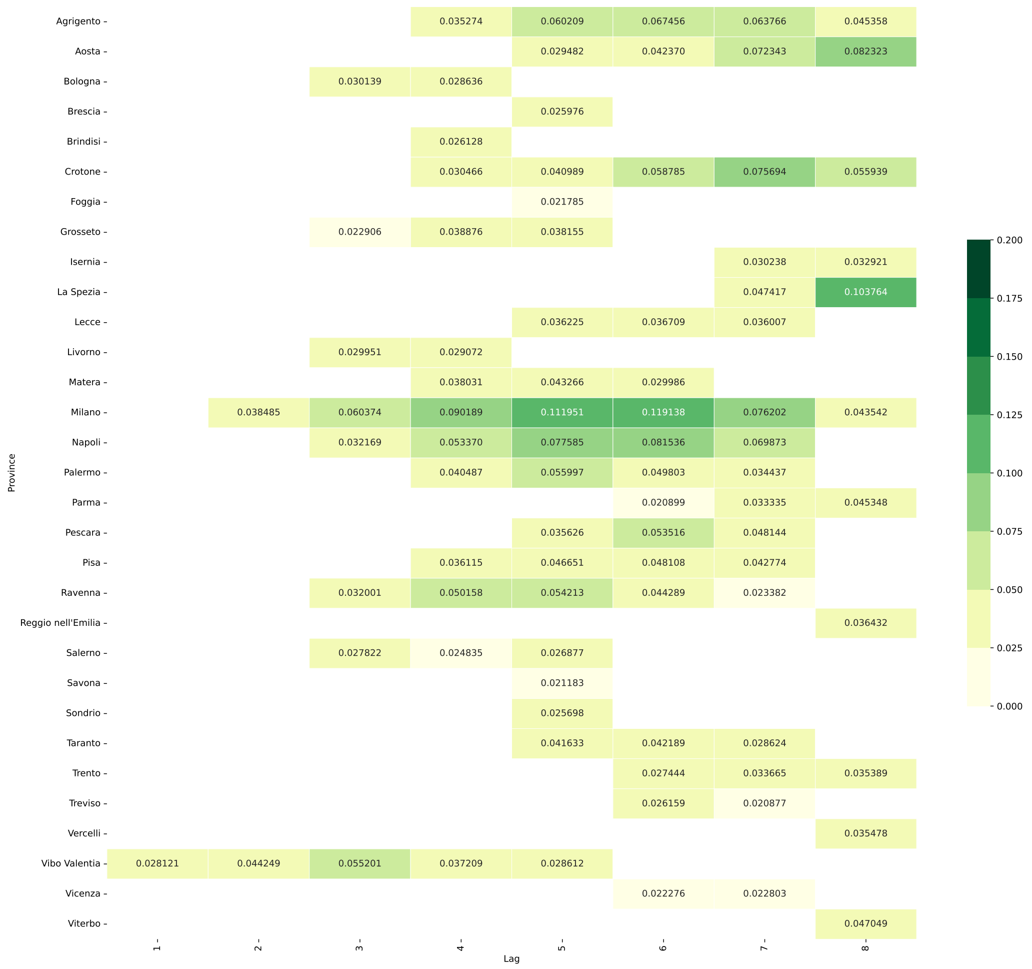

Figure S10: **NETE values from movements to deaths in Italy.** Only statistically significant values are shown (p-value< 0.01).

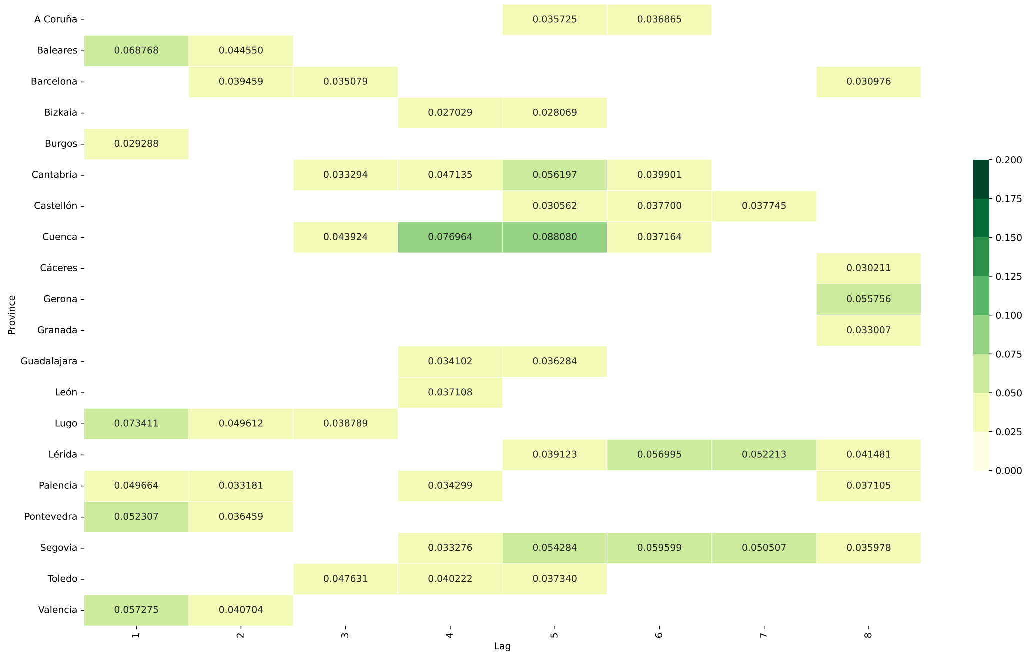

Figure S11: **NETE values from movements to deaths in Spain.** Only statistically significant values are shown (p-value< 0.01).

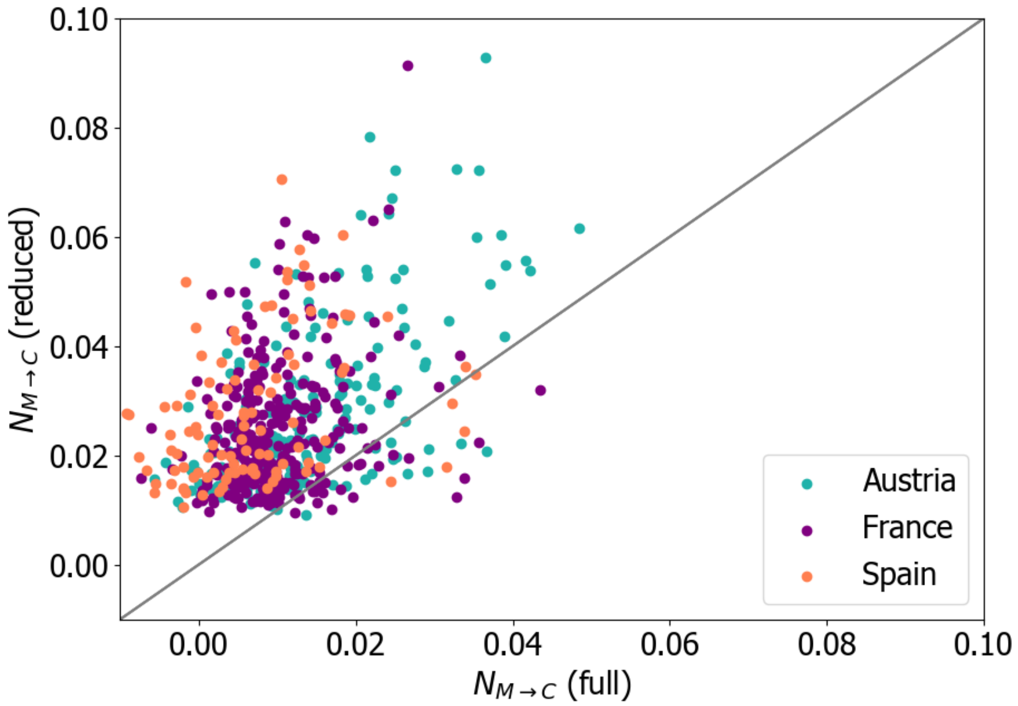

Figure S12: **Comparison of NETE values computed on full time series and reduced time series.** $N_{M\to C}$ computed between time series data collected including the vaccination campaign (full) and not (reduced). The reduced study period ranges from September 1, 2020 to January 31, 2021. The full study period extends up to July 31, 2021. We consider daily time series only to address biases due to small samples.

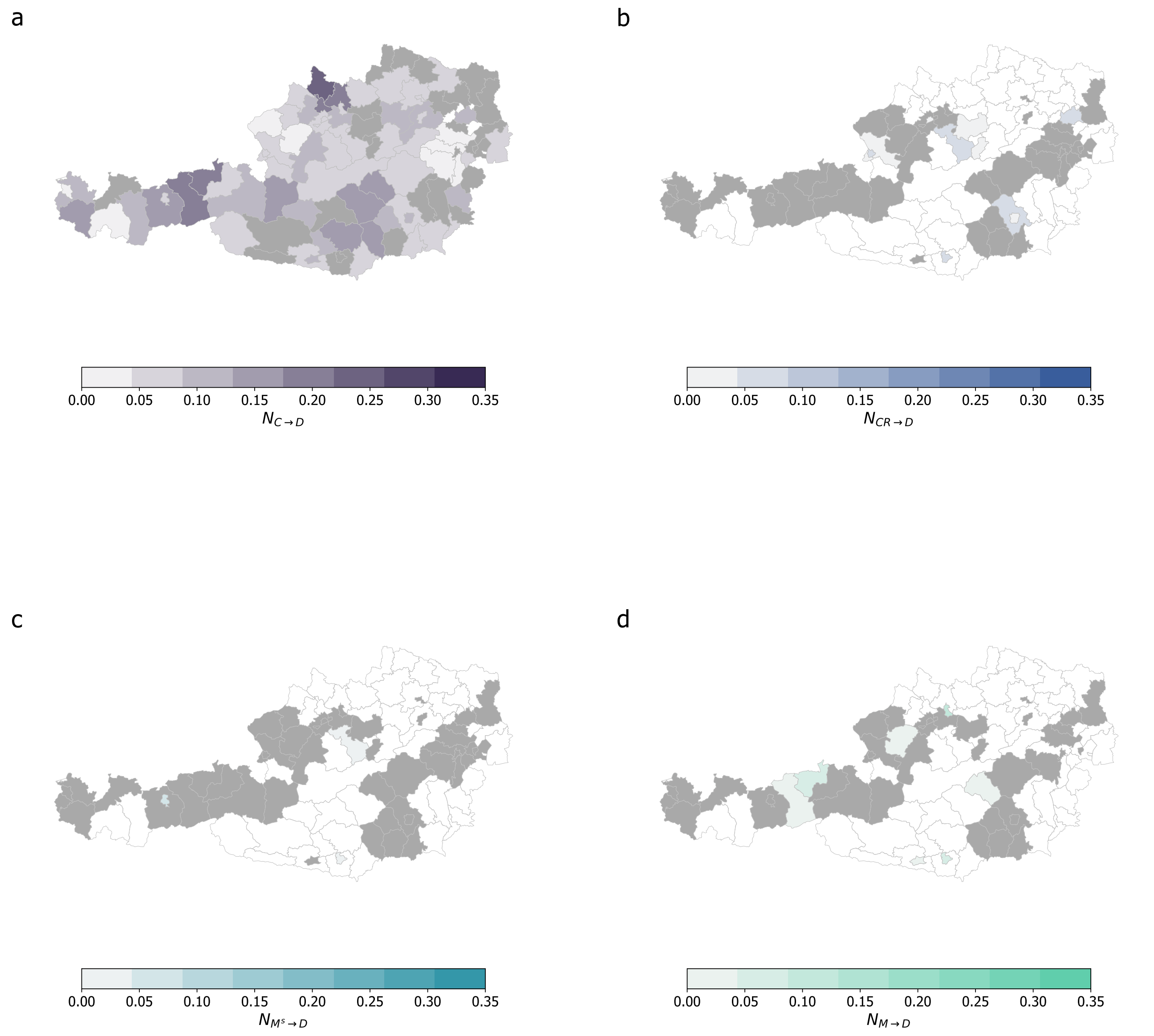

Figure S13: **Spatial variations of normalized effective transfer entropy.** Maps of NETE values computed for different source time series and weekly COVID-19 deaths, in the provinces of Austria: (a) source is COVID-19 cases at lag l=2 weeks, (b) source is contact rate at lag l=7 weeks, (c) source is short-range movement at lag l=7 weeks. (d) source is mid-range movement at lag l=7 weeks. Dark grey indicates provinces with non-significant values of NETE ($p > 0.01$). Provinces in white are excluded from our sample.

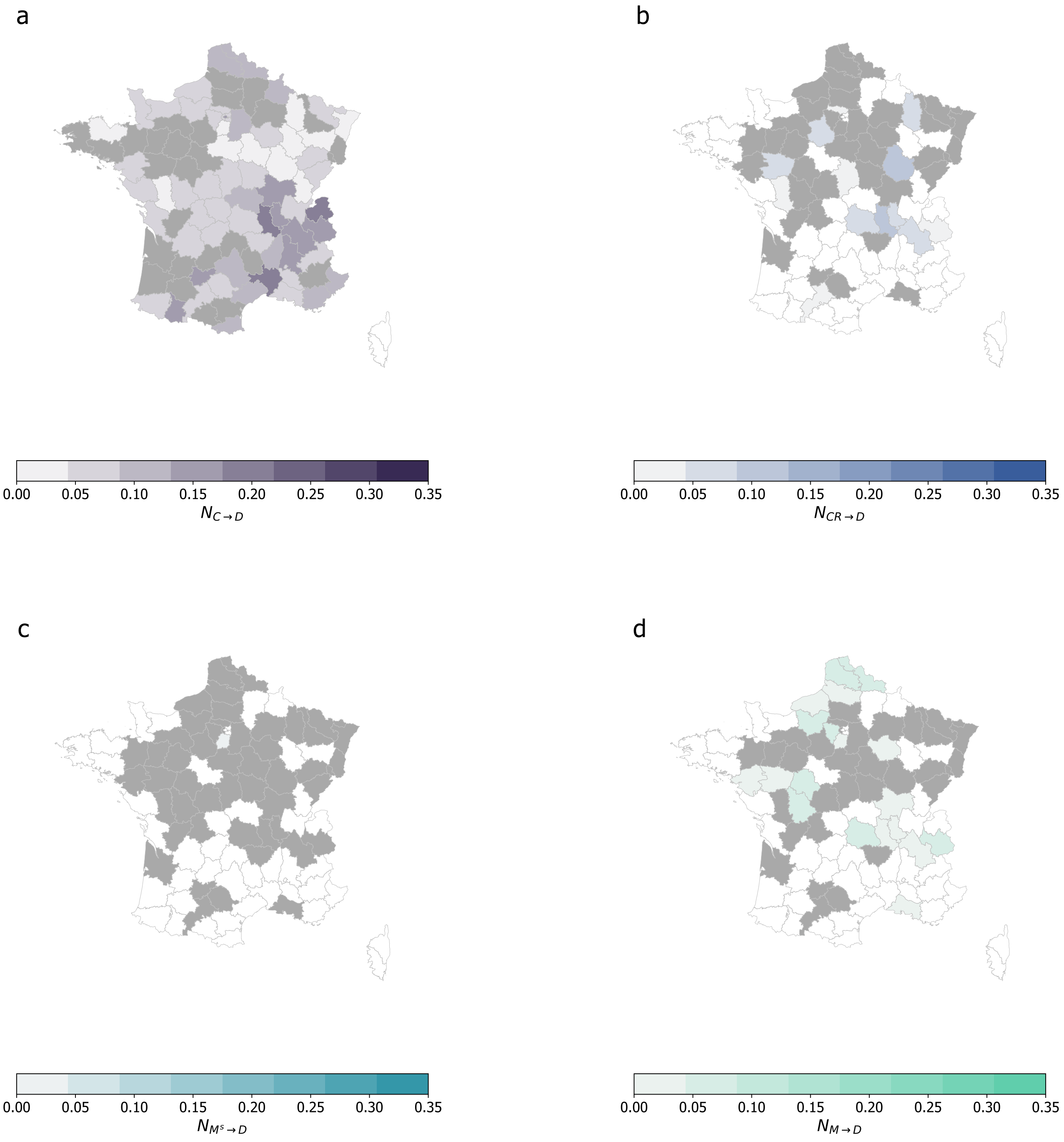

Figure S14: **Spatial variations of normalized effective transfer entropy.** Maps of NETE values computed for different source time series and weekly COVID-19 deaths, in the provinces of France: (a) source is COVID-19 cases at lag l=2 weeks, (b) source is contact rate at lag l=7 weeks, (c) source is short-range movement at lag l=7 weeks. (d) source is mid-range movement at lag l=7 weeks. Dark grey indicates provinces with non-significant values of NETE ($p > 0.01$). Provinces in white are excluded from our sample.

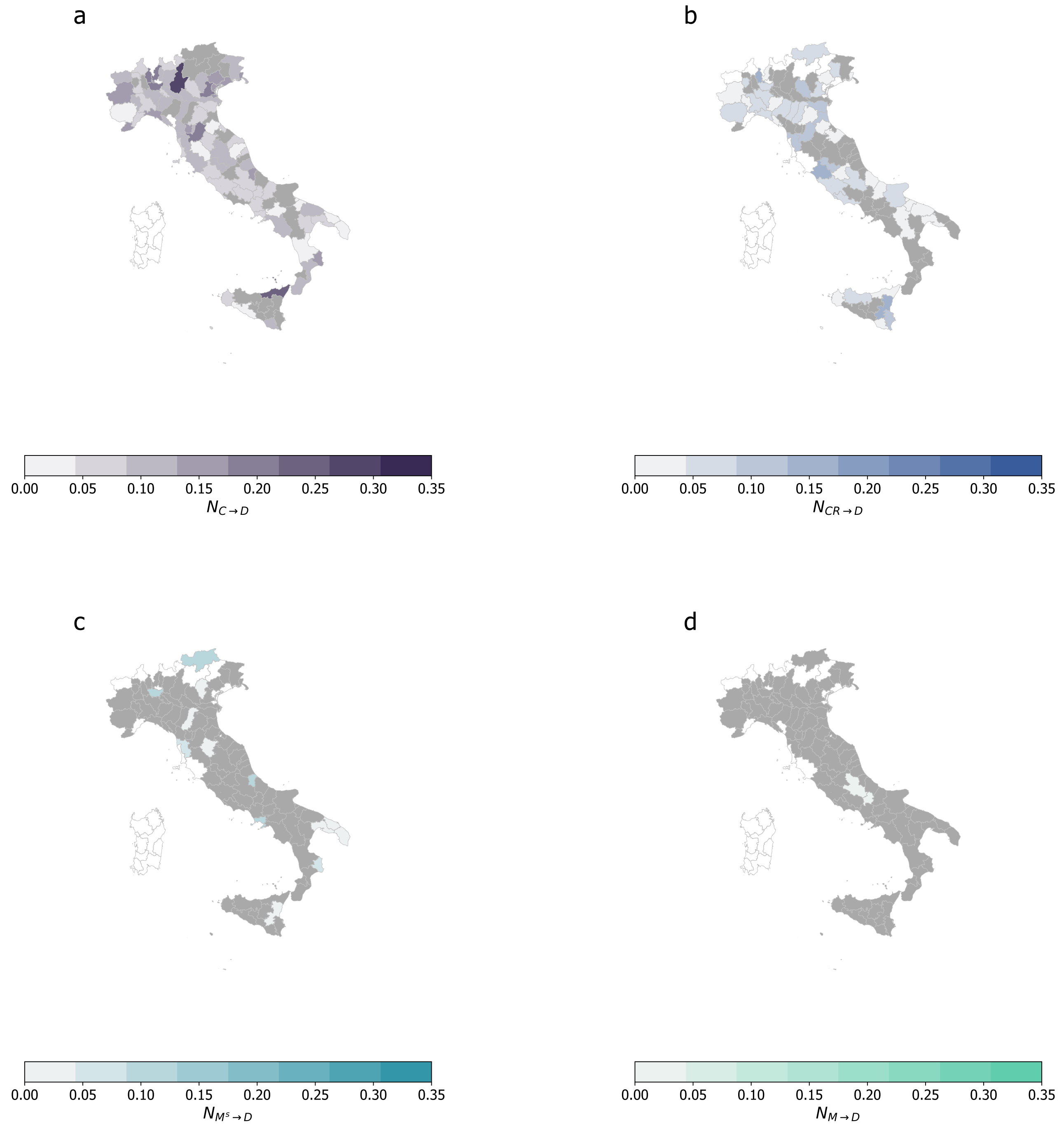

Figure S15: **Spatial variations of normalized effective transfer entropy.** Maps of NETE values computed for different source time series and weekly COVID-19 deaths, in the provinces of Italy: (a) source is COVID-19 cases at lag l=2 weeks, (b) source is contact rate at lag l=7 weeks, (c) source is short-range movement at lag l=7 weeks. (d) source is mid-range movement at lag l=7 weeks. Dark grey indicates provinces with non-significant values of NETE ($p > 0.01$). Provinces in white are excluded from our sample.

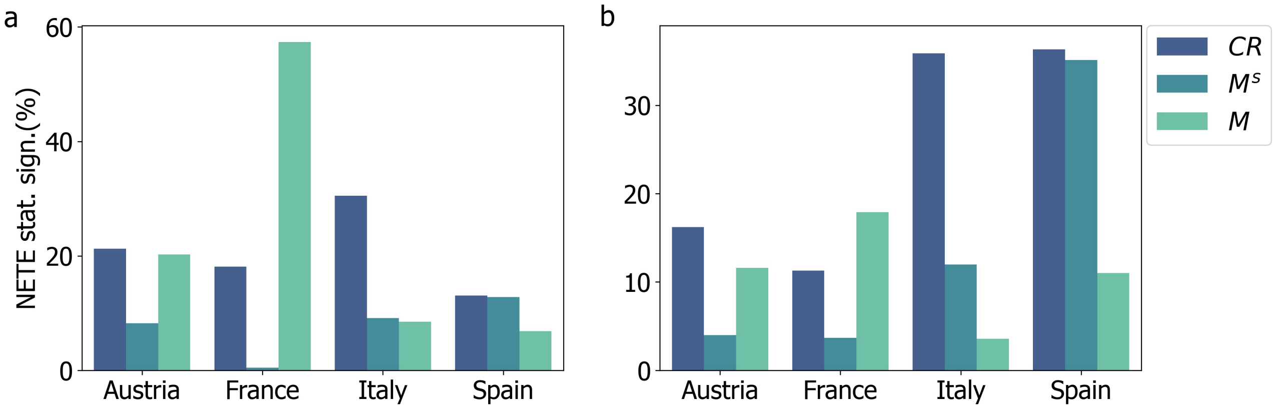

Figure S16: **Percentage of statistically significant NETE values, disaggregated by country and by mobility metric used as source variable.** Target variables are: weekly COVID-19 cases (panel a) and weekly COVID-19 deaths (panel b).

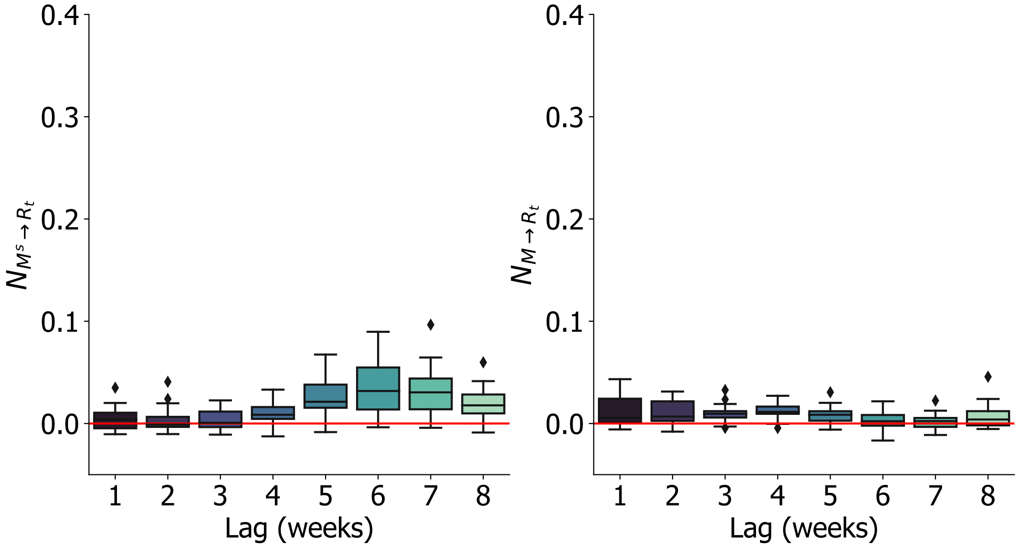

Figure S17: **NETE of mobility indicators and time-varying reproductive number.** Panels show the NETE values computed between mobility time series (short-range mobility M^s and mid-range mobility M) and the time-varying reproductive number R_t. In both cases, the analysis is performed for 18 Italian regions (NUTS2 level) at a weekly scale. Regional R_t values correspond to those officially reported by the Italian Institute of Public Health (ISS) during the period 2020-09-01 to 2021-07-20. The data is publicly available at: `https://github.com/Biuni/rt-italy`.

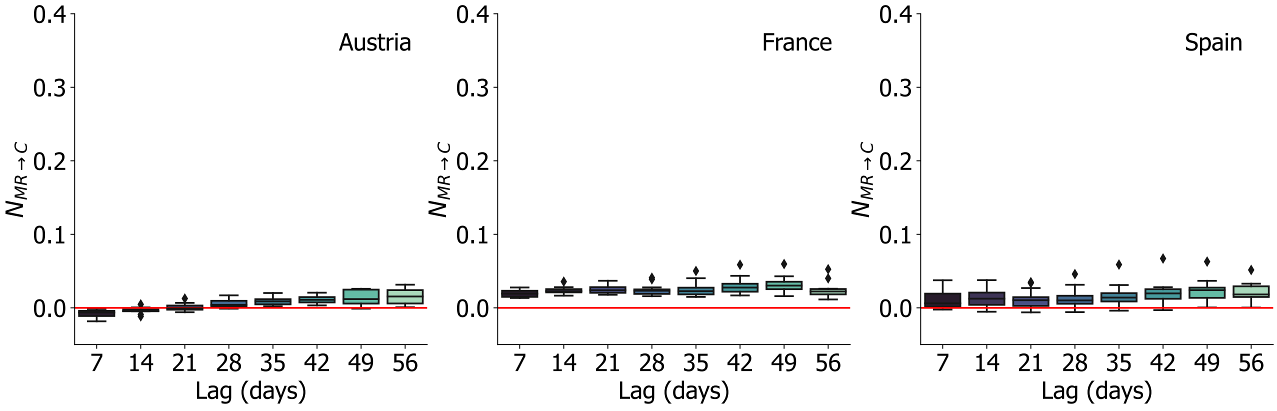

Figure S18: **NETE of relative change in movement and COVID-19 cases.** NETE values computed between the Change in Movement time-series and COVID-19 confirmed cases. The Change in Movement is then defined as the relative change in the average number of tiles visited by Facebook users of a given region with respect to a baseline that predates the beginning of the pandemic. The analysis is performed in all countries at a regional scale (NUTS2 level) and on a daily basis.

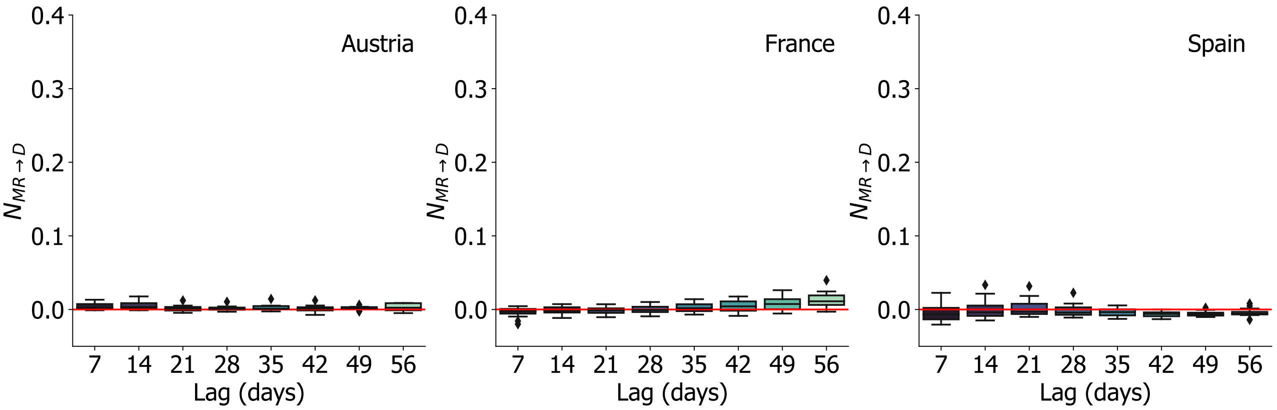

Figure S19: **NETE of relative change in movement and deaths.** NETE values computed between the Change in Movement time-series and COVID-19 confirmed cases. The Change in Movement is defined as the relative change in the average number of tiles visited by Facebook users of a given region with respect to a baseline that predates the beginning of the pandemic. The analysis is performed in all countries at a regional scale (NUTS2 level) and on a daily basis.

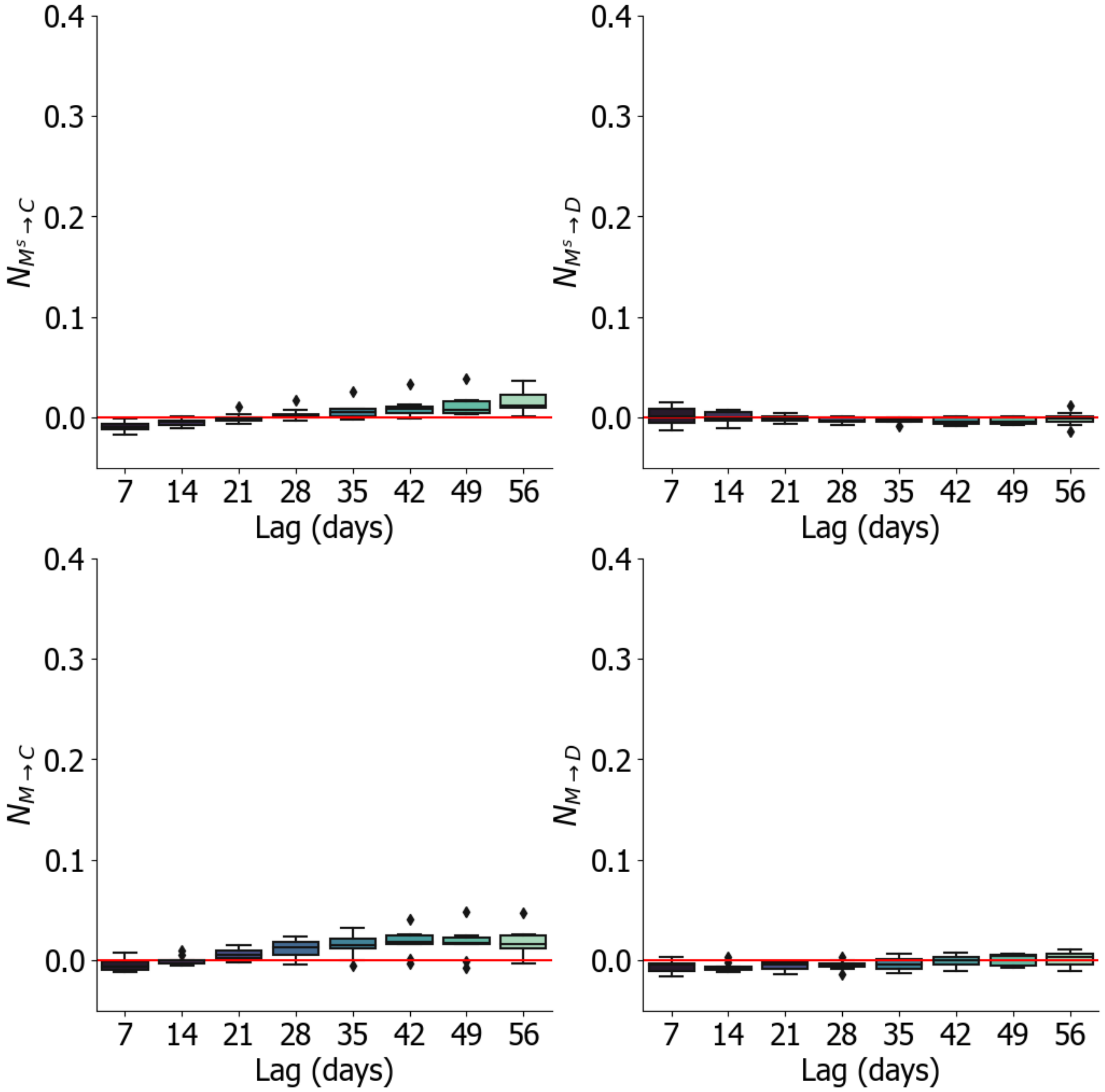

Figure S20: **Effective transfer entropy at NUTS2 level, in Austria.** NETE values computed between short-range (M^s) and mid-range mobility (M), and COVID-19 confirmed cases (C) and deaths (D). The analysis is performed at a regional scale (NUTS2 level) in Austria, and on a daily basis.

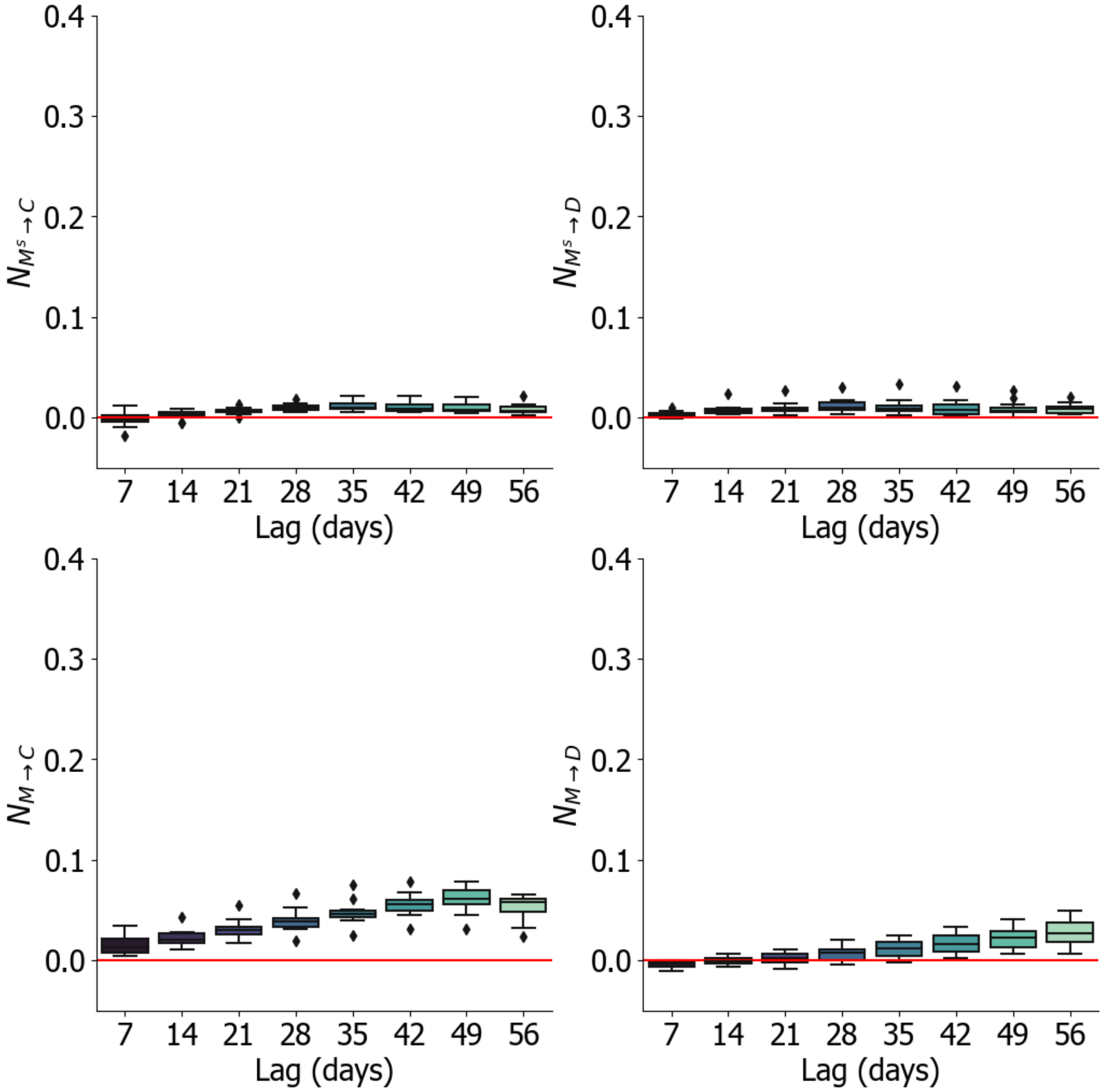

Figure S21: **Effective transfer entropy at NUTS2 level, in France.** NETE values computed between short-range (M^s) and mid-range mobility (M), and COVID-19 confirmed cases (C) and deaths (D). The analysis is performed at a regional scale (NUTS2 level) in France, and on a daily basis.

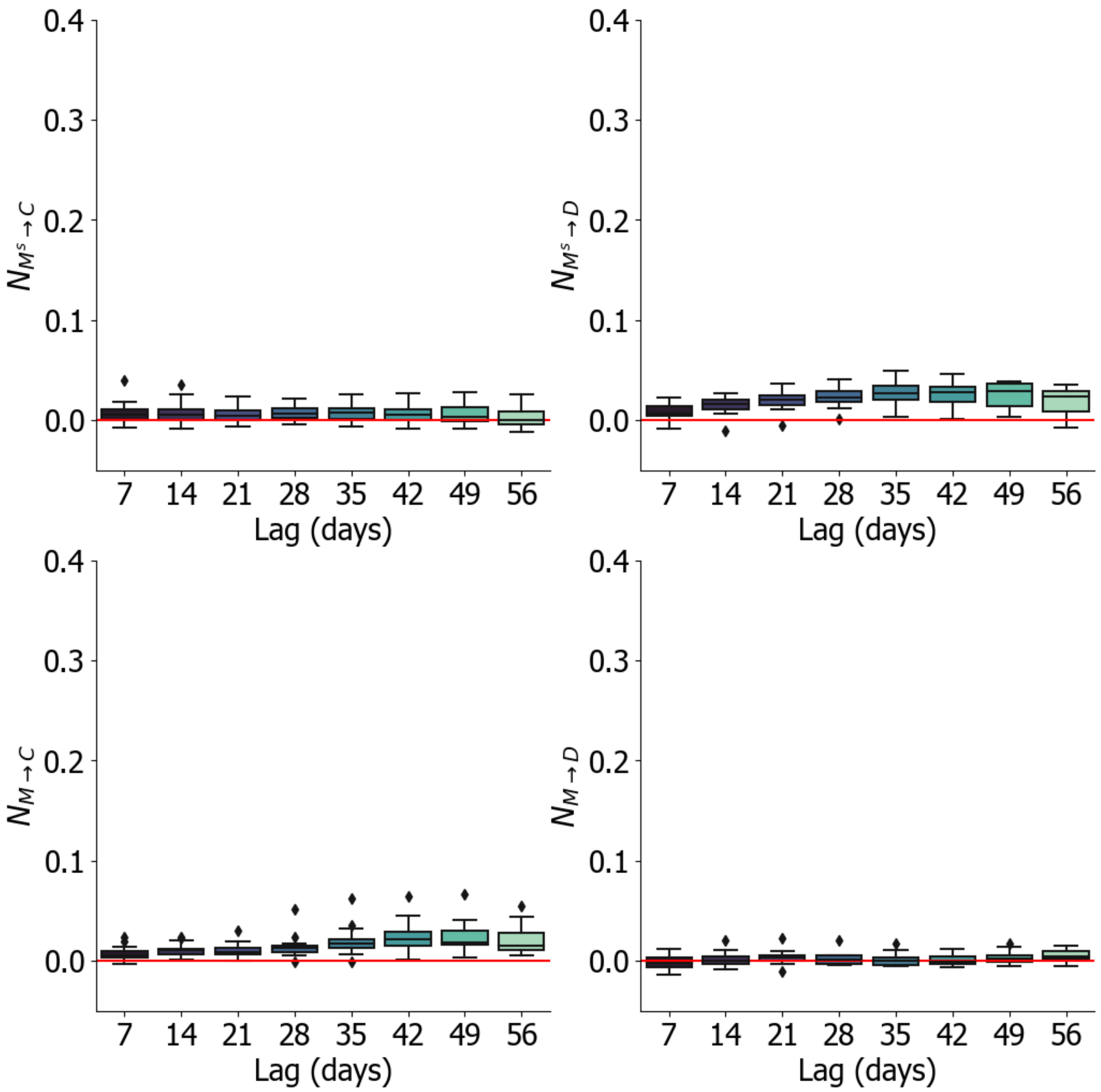

Figure S22: **Effective transfer entropy at NUTS2 level, in Spain.** NETE values computed between short-range (M^s) and mid-range mobility (M), and COVID-19 confirmed cases (C) and deaths (D). The analysis is performed at a regional scale (NUTS2 level) in Spain, and on a daily basis.
